# Childhood Asymmetry Labium Majus Enlargement (CALME): Description of Two Cases

**DOI:** 10.3390/ijerph15071525

**Published:** 2018-07-19

**Authors:** Cristina Salvatori, Ilaria Testa, Marco Prestipino, Maria Elena Laurenti, Sara Riccioni, Giuseppe Di Cara, Nicola Principi, Susanna Esposito, Mirko Bertozzi

**Affiliations:** 1 Paediatric Clinic, Department of Surgical and Biomedical Sciences, Università degli Studi di Perugia, 06132 Perugia, Italy; crisalva_@libero.it (C.S.); ilariatesta@alice.it (I.T.); giuseppe.dicara@unipg.it (G.D.C.); 2 Pediatric Surgery Unit, S. Maria della Misericordia Hospital, 06132 Perugia, Italy; markprestipino@yahoo.it (M.P.); mirkobertozzi@hotmail.com (M.B.); 3 Section of Pathologic Anatomy and Histology, Department of Experimental Medicine, Università degli Studi di Perugia, 06132 Perugia, Italy; anatomia.patologica@ospedale.perugia.it; 4 Radiology Unit, Department of Surgical and Biomedical Sciences, Università degli Studi di Perugia, 06132 Perugia, Italy; sara.riccioni@ospedale.perugia.it; 5 Università degli Studi di Milano, 20122 Milan, Italy; nicola.principi@unimi.it

**Keywords:** childhood asymmetry labium majus enlargement, CALME, vulva

## Abstract

*Background*: Childhood asymmetry labium majus enlargement (CALME) is an uncommon, benign condition that occurs in pre- and early pubertal girls and is characterized by a painless, fluctuating, non-tender labial swelling with normal overlying skin. Recognition of this benign condition is essential. Differentiation with several other diseases that mimic CALME and require different diagnostic and therapeutic approaches is mandatory. Two cases of CALME are described in this report. Differential diagnoses and therapeutic approaches are highlighted. *Case presentation*: The first case was an 11-year-old Caucasian girl referred to our hospital for the evaluation of right labium majus, which showed a palpable, painless, soft, non-tender, non-erythematous enlargement measuring approximately 2 cm with indistinct borders. Ultrasound showed a mass 23 × 18 × 12 mm in diameter. Surgical excision of the mass was performed and in the histopathological evaluation, the tissue specimens were composed of haphazardly arranged vascular channels, adipose tissue and nervous elements that were components of the vulvar soft tissue and were compatible with the diagnosis of CALME. Case 2 was a 6-year-old Caucasian girl who presented a post-traumatic painless mass of left labium majus swelling that progressively increased in volume. Ultrasound study evidenced an ill-defined heterogeneous echotexture mass 26 × 15 × 10 mm in diameter and magnetic resonance imaging confirmed these findings. Histopathological examination was performed after bioptic sampling evidencing normal constituents of vulvar soft tissue, including fibroblast, collagen, adipose tissue, blood vessels and nerves compatible with CALME. *Conclusions*: CALME is a particular clinical condition that occurs mainly in pre-pubertal girls and has a benign course but poses numerous problems in differential diagnosis that can be solved only with careful clinical observation and with a careful use of radiological imaging techniques. Our cases, in agreement with recent literature, suggest that radical excision is not recommended and that surgical biopsy should be taken into consideration only in cases of doubt.

## 1. Background

Childhood asymmetry labium majus enlargement (CALME) is an uncommon, benign condition that occurs in pre- and early pubertal girls and is characterized by a painless, fluctuating, non-tender labial swelling with normal overlying skin [1]. The lesion has plump appearance and soft consistency. Histologically, the main finding is a significant proliferation of fibrous tissue surrounding normal vulvar elements, such as adipose tissue, blood vessels, and nerves. Fibroblasts do not have pleomorphism or mitotic activity, and no capsule or margin is detected. Moreover, as in the normal vulvar tissue, estrogen receptors are evidenced [1]. Finally, sonography confirms labial enlargement without a definable mass, and magnetic resonance imaging (MRI) shows ill-defined T1-weighted hypointense signal and hypo- to isointense to muscle on T2-weighted images [2]. 

The term CALME was coined in 2005 by Vargas et al. to describe a disease with the previously cited characteristics, but it is highly likely that it was discovered some years before, although identified with different names [3]. Cases resembling CALME have been reported as hamartoma in 2000 [4] and as fibroma in 2004 [5]. Moreover, even later than 2005, other CALME cases have been reported and named as fibrous hyperplasia [6] or lipoma [7]. These factors explain why the real incidence of CALME is not precisely defined. However, recognition of this benign condition is essential. Differentiation with several other diseases that mimic CALME and require different diagnostic and therapeutic approaches is mandatory. Moreover, when diagnosed, CALME treatment has to be attentively evaluated and has to remain as conservative as possible. Two cases of CALME are described in this report. Differential diagnoses and therapeutic approaches are highlighted. 

## 2. Case Presentation

Children’s parents gave their written informed consent for management of their daughter and for publication of the clinical case. Ethics Committee of Santa Misericordia Hospital in Perugia, Italy, was informed about management and publication of these two cases (project identification code n. WAidid2017_02).

### 2.1. Case 1 

An 11-year-old Caucasian girl was referred to our hospital ward for the evaluation of right labium majus swelling. History of illness, systemic disease or trauma was denied. On admission, her vital parameters were normal. In her physical examination, there was no abnormality except for the right labium majus, which showed a palpable, painless, soft, non-tender, non-erythematous enlargement measuring approximately 2 cm with indistinct borders. The vaginal introitus and external meatus were normal, with no evidence of clitoral hypertrophy. Laboratory investigations revealed normal complete blood count, liver and renal function tests. Serum levels of acute phase reactants were within normal limits, such as serum levels of FSH, LH, estradiol, total testosterone, and thyroid hormones. Ultrasound showed a mass 23 × 18 × 12 mm in diameter characterized by an increased amount of labial soft tissue on the affected side with a similar echogenicity to the contralateral side.

**Figure 1:**
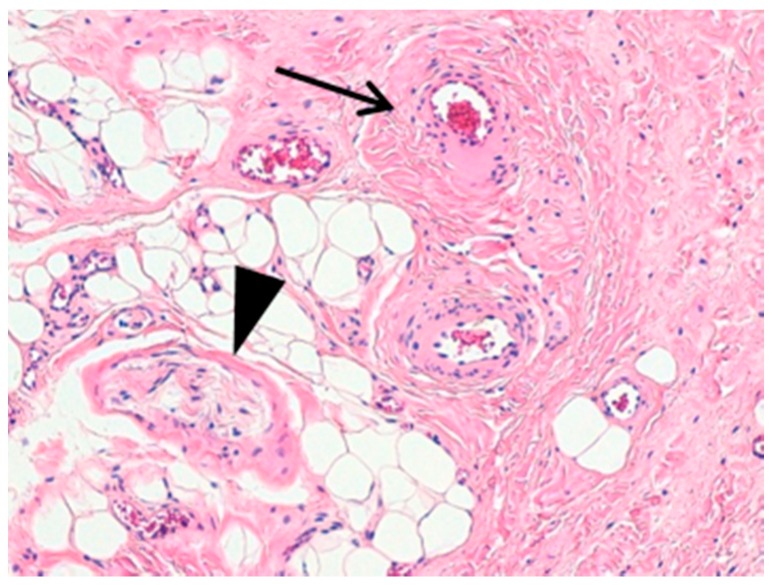
(Hematoxylin-Eosin, original magnification 100×). Different fields of the same slide showing connective and adipose tissue intermingled with small blood vessels (large arrow) and nerves (arrowhead). All these structures, although normally located in this anatomic area, grew up in a disorganized fashion.

Surgical excision of the mass was performed. In the histopathological evaluation, the tissue specimens were composed of haphazardly arranged vascular channels, adipose tissue and nervous elements that were compatible with the diagnosis of CALME (Figure 1). However, all these components are usually constituents of the normal vulvar soft tissue.

Follow-up was performed at 1, 6 and 12 months without evidence of recurrence.

### 2.2. Case 2 

**Figure 2:**
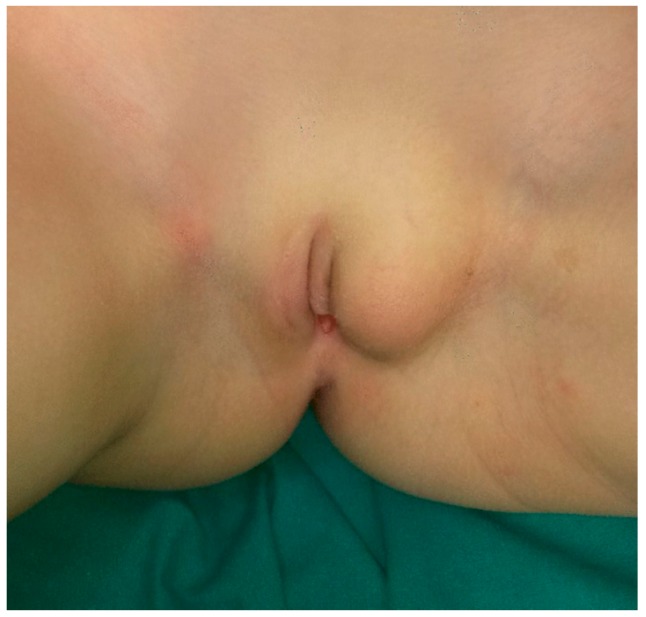
Clinical presentation of the left labius majus swelling in a pre-pubertal female.

A 6-year-old Caucasian girl presented a post-traumatic painless mass of left labium majus swelling that progressively increased in volume (Figure 2). 

**Figure 3:**
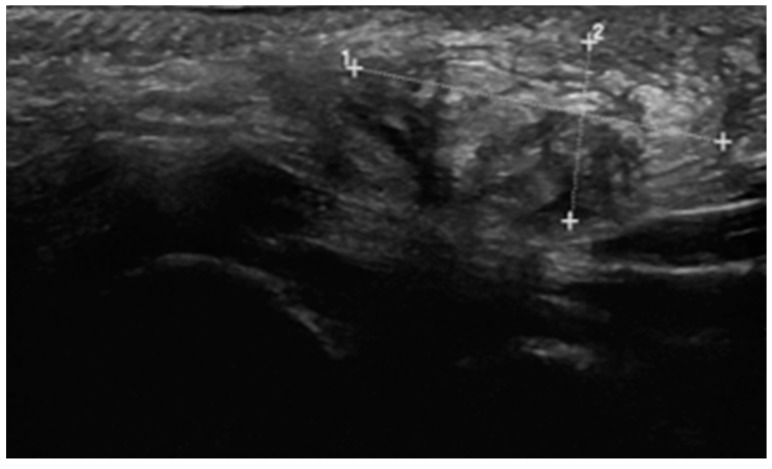
Sonographic findings evidenced a mass of 26 × 15 × 10 mm of diameters with heterogeneous echotexture.

A careful clinical examination was made, with no evidence of other alterations. As in Case 1, laboratory tests revealed no signs of a chronic or neoplastic condition and no endocrine abnormalities. Ultrasound study evidenced an ill-defined heterogeneous echotexture mass 26 × 15 × 10 mm in diameter (Figure 3). 

The area of enlargement blended into the normal labial tissue, and there was no definable capsule. Magnetic resonance imaging (MRI) confirmed these findings, namely, asymmetrical mildly enlarged labial tissue composed of homogeneous hypointense signal on T1-weighted imaging and hypo- to isointense to muscle on T2-weighted images. 

**Figure 4:**
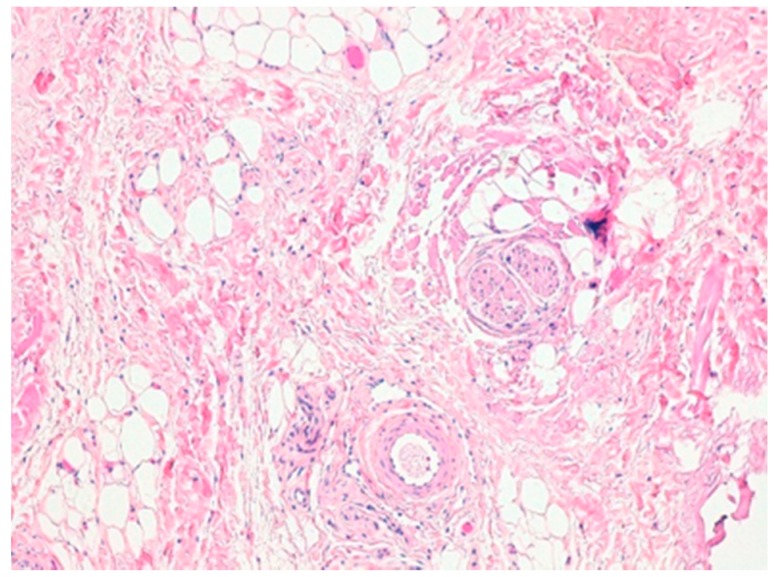
(Hematoxylin-Eosin original magnification 100×). Mixing of fibrous and adipose tissue, blood vessels and nerves.

Histopathological examination was performed after bioptic sampling evidencing normal constituents of vulvar soft tissue, including fibroblast, collagen, adipose tissue, blood vessels and nerves compatible with CALME (Figure 4). The immunohistochemistry results were positive for estrogen and progesterone receptors. No evidence of recurrence was found at follow-up visits performed at 1 and 6 months after surgical excision. 

## 3. Discussion

CALME is a clinical condition whose origins are unknown. The most accepted hypothesis considers CALME to be the effect of a hormonal response, as occurs during breast development. The vulva is responsive to sex hormones and, at puberty, it undergoes profound changes. The labia become gradually larger, thicker, and wrinkled [8]. Asymmetric breast swelling at puberty is common [9], and it is thought that the same can occur for the labia [1]. The evidence that fibroblasts detected in the expansion of the labium majus are frequently immunohistochemically positive for estrogen and progesterone receptors supports this hypothesis [1].

The two cases reported here have clinical, radiological, and histological findings that strongly support the diagnosis of CALME. Diagnosis was suspected based on clinical signs and symptoms and sonography/MRI results and was later confirmed by histologic findings. However, several differential diagnoses were considered before deciding to perform confirmatory tissue biopsy; mimicking lesions, inguinal hernia, vascular malformations, lipoma, neurofibroma, rhabdomyosarcoma, angiofibroblastoma, Bartholin’s duct cyst or abscess, and labial hypertrophy were also taken into account. Due to its fluctuating nature, CALME should be considered in a differential diagnosis with inguinal hernia [10], but in these cases this diagnosis was excluded by the finding that fluctuation was significantly less evident than that usually detected in hernia cases and by radiological characteristics of the palpable mass. Vascular malformations were excluded because of the lack of both bluish discoloration of the skin and a history of pain and thrombosis, as well as the evidence of no varicosities, cystic spaces or homogenous solid appearance at sonography/MRI [10]. Diagnoses of lipoma and neurofibroma were considered poorly consistent with clinical findings, as both of these lesions present as well-defined masses on palpation [11]. Bartholin’s duct diseases are exceptional in pre-pubertal girls [12]. Labial hypertrophy is bilateral and occurs in late or post-pubertal subjects, in contrast to CALME, which is unilateral in the greatest majority of the cases and occurs earlier [1]. 

Confirmation of the diagnosis is usually based on biopsy and histological evaluation of the enlarged tissue. For therapy, when CALME is described, surgical excision is considered the best solution [1]. In both these cases, surgical removal was decided in the fear that, with puberty, the mass would increase significantly and lead to esthetical problems or deviation of the urinary stream. However, recently, a more conservative approach to therapy has been suggested, provided that accurate follow-up, including sonography, is possible [13]. Because CALME is a benign disease [1,2,3,4,5,6,7,8,9,10,11,12,13,14], a number of CALME cases can spontaneously reduce [1], and radical excision can lead to disfigurement [4]. Moreover, excision can be poorly effective and can be associated with recurrence in 50% of the cases [1].

## 4. Conclusions

CALME is a particular clinical condition that occurs mainly in pre-pubertal girls and has a benign course but poses numerous problems in differential diagnosis that can be solved only with careful clinical observation and with a careful use of radiological imaging techniques. Our cases, in agreement with recent literature, suggest that radical excision is not recommended and that surgical biopsy should be taken into consideration only in cases of doubt.
